# Chronic virus infections supress atopy but not asthma in a set of children from a large latin american city: a cross-section study

**DOI:** 10.1186/1471-2466-11-24

**Published:** 2011-05-14

**Authors:** Rafael V Veiga, Sergio S Cunha, Vitor CC Dattoli, Álvaro C Cruz, Phillip J Cooper, Laura C Rodrigues, Maurício L Barreto, Neuza M Alcantara-Neves

**Affiliations:** 1 SCAALA (Social Change, Asthma and Allergy in Latin America) Research Program; 2 Departamento de Ciências da Biointeração, Instituto de Ciências da Saúde, Universidade Federal da Bahia, Av. Reitor Miguel Calmon, Canela, Salvador, CEP 40110100, Brazil; 3 Departamento de Medicina Social, Centro de Ciências da Saúde, Universidade Federal de Pernambuco, Av. Prof. Moraes Rêgo, 1235, Cidade Universitária, Recife, CEP 50670-901, Brazil; 4 ProAR, Departamento de Clínica Médica, Faculdade de Medicina da Bahia, Universidade Federal da Bahia, Av. Reitor Miguel Calmon, Canela, Salvador, CEP 40110100, Brazil; 5 Colegio de Ciencias de la Salud, Universidad San Franscisco de Quito, Hospital de Los Valles, Via Interoceanica Km 12.5, Quito, Ecuador; 6 Molecular and Biochemical Parasitology, Liverpool School of Tropical Medicine, Pembroke Place, Liverpool L3 5QA, UK; 7 London School of Hygiene and Tropical Medicine, Keppel Street, London WC1E 7HT, UK; 8 Instituto de Saúde Coletiva, Universidade Federal da Bahia, Rua Basílio da Gama S/N Canela, Salvador, CEP 40.110-040, Brazil

## Abstract

**Background:**

The prevalence of allergic diseases has increased over recent decades in affluent countries, but remains low in rural populations and some non-affluent countries. An explanation for these trends is that increased exposure to infections may provide protection against the development of allergy. In this work we investigated the association between exposure to viral infections in children living in urban Brazil and the prevalence of atopy and asthma.

**Methods:**

School age children living in poor neighborhoods in the city of Salvador were studied. Data on asthma symptoms and relevant risk factors were obtained by questionnaire. Skin prick tests (SPTs) were performed to seven aeroallergens, and specific IgE was measured to four of these. Viral infections were determined by the presence of specific IgG in serum to Herpes simplex (HSV), Herpes zoster (HZV), Epstein-Barr (EBV), and Hepatitis A (HAV) viruses.

**Results:**

A total of 644 (49.7%) children had at least one allergen-specific IgE> 0.35 kU/L and 489 (37.7%) had specific IgE> 0.70 kU/L. A total of 391 (30.2%) children were skin test positive (SPT+), and 295 (22.8%) children were asthmatic. The seroprevalence of viral infections was 88.9% for EBV, 55.4% for HSV, 45.5% for VZV and 17.5% for HAV. Negative associations were observed between SPT+ and HSV (OR = 0.64, CI = 0.51, 0.82) and EBV (OR = 0.63, CI = 0.44, 0.89) infections, but no associations were seen between viral infections and the presence of allergen-specific IgE or asthma.

**Conclusion:**

These data do not support previous data showing a protective effect of HAV against atopy, but did show inverse associations between SPT+ (but not specific IgE+) and infections with HSV and EBV. These findings suggest that different viral infections may protect against SPT+ in different settings and may indicate an immunoregulatory role of such infections on immediate hypersensitivity responses. The data provide no support for a protective effect of viral infections against asthma in this population.

## Background

The prevalence of allergic diseases, such as rhinitis, asthma and eczema has increased in affluent countries over recent decades, and may have increased also in cities of non-affluent countries such as in Latin America [1]. Such temporal trends occurring over a relatively short period of time are unlikely to be explained by changes in genetic susceptibility and are most likely explained by changes in environmental exposures such as those associated with the adoption of a modern or 'westernized' lifestyle [2].

Strachan [3] observed that children lower in the birth order had a reduced prevalence of hay fever and interpreted this observation in terms of younger children being more highly exposed to infections in early life through contact with their older siblings. This interpretation, coined the hygiene hypothesis, and has been widely promoted to explain the temporal trends in allergic disease prevalence.

Allergic diseases are complex inflammatory disorders with significant genetic components [4,5] and are strongly influenced by environmental factors [6-8]. Allergic inflammation is considered to develop following immediate hypersensitivity reactions to environmental antigens which leads to the development of an immune response characterized by high levels of IgE and increased numbers of eosinophils and mast cell cells, and a Th2 immune profile [9]. In the early 1990 s, with the discoveries of Th1 and Th2 lymphocyte populations in animal models and the realization that infection with bacteria and viruses induce the innate immune system to release Th1 cytokines, a possible biological explanation for the hygiene hypothesis emerged. When born, a child has a predominantly Th2 immune response, requiring the presence of infections to stimulate the Th1 system in early childhood and achieve a balance between Th1 and Th2 responses to prevent a Th2 bias that favor Th2 allergic diseases [10]. Although humans may respond differently to infections, there is epidemiological evidence for inverse associations between infections and allergic diseases that support this hypothesis. Such infections include hepatitis A [11], Herpes simplex [12] and Epstein-Barr viruses [13], viral lower respiratory tract infections [14], and *Mycobacterium tuberculosis *infection [15].

To test the hypothesis that childhood viral infections protect against allergy and asthma, we compared the seroprevalence of common childhood viral infections between children with and without atopy and asthma living in poor neighborhoods in a Brazilian city.

## Methods

### Population and study design

The study was conducted in a cohort of 1,445 children living in poor neighborhoods of Salvador, a city with a population of 2.5 million with a high prevalence of asthma [16]. This cohort of children aged 4-13 years was derived from an earlier study on the impact of sanitation on childhood diarrhea conducted in 24 sentinel areas in Salvador, and the study design is described in detail elsewhere [17]. A questionnaire to collect information on risk factors, demographic factors, and key allergic symptoms [ISAAC-based] was administered to the child's parent or guardian in 2005. The children underwent skin prick tests (SPTs) to seven aeroallergens. Blood samples were collected and the sera were stored at -20°C and used to measure IgG to relevant pathogens and allergen-specific IgE (sIgE).

This study was approved by the Ethics Committee for Research of the Instituto de Saúde Coletiva da Universidade Federal da Bahia and CONEP (National Council of Ethics in Research); written consent to participate in the work, containing information about history of allergies and their risk factors, was obtained from parents or guardians.

### Asthma definition

Children were classified as having asthma if parents reported wheezing in the previous 12 months and at least one of the following: (i) diagnosis of asthma ever; (ii) wheezing with exercise in the last 12 months; (iii) 4 episodes wheezing in the last 12 months; (iv) waking up at night because of wheezing in the last 12 months.

### Skin prick test

The SPT was carried out using ALK-Abello reagents (São Paulo, Brazil) for the following allergens identified as regionally relevant by previous unpublished work: *Dermatophagoides pteronyssinus, Blomia tropicalis, Blattella germanica, Periplaneta americana*, dog and cat epithelia, and a mixture of fungi (*Aspergillus amstelodami, A. fumigatus, A. niger, A. terrus, Penicillium brevicompactum, P. expansum, P. notatum, P. roqueforti, Cladosporium fulvum*, and *C. herbarum*). Positive and negative controls were 10 mg/mL histamine and saline, respectively. Allergens and controls were applied to the right forearm. After 15 minutes, the reaction was measured and was considered positive when the mean wheal diameter was at least 3 mm greater than the negative control. Children positive for at least one of the studied allergens were classified as SPT positive. Children who used anti-allergic drugs were excluded from the study.

### Measurements of anti-aeroallergen IgE antibodies

The presence of specific IgE antibodies to *D. pteronyssinus, B. tropicalis, P. americana *and *B. germanica *in the children's sera was determined using the lmmunoCap system (Phadia Uppsala, Sweden). These antigens were chosen because they were the most frequently observed to cause SPT reactivity in these children. The results, expressed in kU/L (lower limit corresponding to 2.4 ng/ml), were obtained by a standard curve produced by serial dilutions of human IgE against a standard serum IgE provided by the World Health Organization (WHO standard 75/502). The IgE cut preconized by the manufacturer is 0.35 kU/L. However cross-reactivities between allergen reactive IgEs and many organisms, including helminthes have being reported at this cut-off [18]. That is why we decided to use both IgE ≥0.35 kU/L and ≥0.70 kU/L cut-offs. Children were considered positive when they had values greater than these cut-off points for at least one of the studied allergens.

### Detection of viral IgG

The presence of serum antibodies in the children's sera against herpes simplex (HSV), herpes zoster (HZV) and Epstein-Barr (EBV) viruses was determined by ELISA assay using kits from Diamedix (Miami, Florida, USA), following the manufacturer's instructions. For the hepatitis A virus (HAV), kits from ADALTIS were used (Toronto, Canada). The cut-off points were determined by the index derived from the relationship: absorbance of sample/calibrator absorbance (solution containing human serum or defibrinated plasma weakly reactive for antibodies against the respective virus and 0.1% sodium azide). Indices greater than 1.1 were considered positive, less than 0.9 were considered negative and between 1.1 and 0.9 were considered indeterminate, and these children were excluded from the analysis.

### Statistical analysis

Analyses were done with children with no missing data for the variables analyzed, and comparisons were made between the studied and the excluded groups using the chi-square test or the *t*-test (two-tailed). We assessed the association between the presence of serum antibodies for the 4 viruses (main exposures) and specific serum IgE, skin reactivity for environmental allergens and the presence of asthma (outcome variables). Potential confounders for the association between virus infections and outcomes were: maternal education, presence of a sewage disposal system, frequency of changing bed linen, number of siblings, presence of cat and dog in the house, smoking parents, presence of mold or dampness in the walls of the house (by inspection) and if the child had attended daycare. Although we collected data about children vaccination, none was vaccinated for the viruses used in this analysis, and then, this variable was removed from the study.

The associations between exposure outcomes (IgE, SPT and asthma) were assessed using logistic regression to estimate odds ratios (OR) and 95% confidence intervals (CIs). Variables were entered individually in multivariate models and if the variable was non-significant (P > 0.05) it was removed, but if significant, was kept in the analysis. Only significant variables were kept in the models. Gender and age were considered *a priori *confounders and kept in all models. Analysis on multiplicative interactions between each infection and gender in relation to all outcomes was done, and co-linearity between viral infections was also assessed. Finally, we tested for the effects of clustering by neighborhood, using random effects models, but because such adjustment did not alter effects, results without adjustment for clustering were provided. Analyses were performed using SPSS (Release 16.0, SPSS, Inc) and multinomial (polytomous) logistic regression analyses using STATA (Release 9.0, Stata Corp).

## Results

### Characteristics of study population

Of the 1,445 children enrolled in the cohort, 89.7% (1296) with complete data were included in the present analysis. No significant differences were found between the 149 excluded and the 1,296 studied included within respect to relevant baseline variables. The mean age of the children was 7.2 years (standard deviation (SD = 1.7), and 53.8% were male. A history of parental asthma was present in 13.3% of the children, and 27.4% had at least one smoker at home. The results showed that 44.2% of the children had the bed linen changed more than once a week, 82.8% had a sewage system in their home, 64.9% had mold and moisture in the walls of their houses, and 39.6% lived with dogs and 17.7% with cats. Only 386 (29.8%) of the children had mothers who had completed secondary education. Children had, on average, 1.8 (SD = 1.8) siblings. Only 209 (16%) children had attended daycare. Seropositivity for the studied viruses was 88.9% for EBV, 55.4% for HSV, 45.5% for VZV and 17.5% for HAV. Seroprevalence increased with age, and only herpes simplex was found to be associated with gender (greater in girls; data not shown). The prevalence of IgE-sensitization using the two cut-offs were: IgE> 0.35 kU/L, and IgE> 0.70 kU/L, 49.7% and 37.7%, respectively. SPT positivity for at least one of the studied allergens was present in 30.2% of the children, and the prevalence of asthma was 22.8% (Table 1).

**Table 1:** Associations between viral infections with aeroallergen IgE, skin test reactivity (SPT) and asthma

Variables N = 1296	Total population n = 1296	Number and percentage of children positive for the outcomes					
		
		IgE ≥ 0.70 n = 489 (37.7%)		SPT n = 391 (30.2%)		Asthma n = 295 (22.8%)	
	n (%)	n (%)	p-value	n (%)	p-value	n (%)	p-value
**Gender**							

Male	697(53.8)	*306(43.9)	**<0.001**	**233(33.4)**	**0.006**	164(23.5)	0.477
**Age (years)**							
4-6	333(25.7)	123(36.9)	0.914	92(27.6)	0.230	**113(33.9)**	**<0.001**
6 and 7	531(41.0)	200(37.7)		156(29.4)		**117(22.0)**	
7-11	432(33.3)	166(38.4)		143(33.1)		**65(15.0)**	
**Parental asthma**	173(13.3)	57(32.9)	0.163	50(28.9)	0.696	**61(35.3)**	**<0.001**
**Sibling n^o^**.							
0	241(18.6)	98(40.7)	0.683	78(32.4)	0.213	54(22.4)	0.936
1	459(35.4)	167(36.4)		148(32.2)		109(23.7)	
2	306(23.6)	118(38.6)		91(29.7)		67(21.9)	
3 or more	290(22.4)	106(36.6)		74(25.5)		65(22.4)	
**Mother's education**							
Illiterate	177(13.7)	74(41.8)	0.616	50(28.2)	0.570	54(30.5)	**0.025**
Primary complete	444(34.3)	160(36.0)		126(28.4)		102(23.0)	
Secondary incomplete	289(22.3)	109(37.7)		90(31.1)		66(22.8)	
Secondary complete	386(29.8)	146(37.8)		125(32.4)		**73(18.9)**	
**Daycare ever **Yes	208(16.0)	77(37.0)	0.817	53(25.5)	0.108	**61(29.3)**	**0.014**
**Smoker at home**	355(27.4)	127(35.8)	0.372	99(27.9)	0.272	88(24.8)	0.285
**Sewage system **Yes	1073(82.8)	414(38.6)	0.165	327(30.5)	0.599	242(22.6)	0.694
**Change bed linen1 × week**	573(44.2)	212(37.0)	0.628	173(30.2)	0.988	137(23.9)	0.381
**Cat at home **Yes	230(17.7)	83(36.1)	0.570	61(26.5)	0.184	**66(28.7)**	**0.018**
**Dog at home **Yes	513(39.6)	187(36.5)	0.442	147(28.7)	0.336	129(25.1)	0.098
**Mold/moisture at home**	841(64.9)	318(37.8)	0.935	253(30.1)	0.926	**210(25.0)**	**0.010**
**Seropositivity**							
***Herpes simplex***	712(54.9)	254(35.7)	0.092	**185(26.0)**	**<0.001**	158(22.2)	0.588
***Varicella zoster***	588(45.4)	216(36.7)	0.500	171(29.1)	0.437	132(22.4)	0.806
***Epstein-Barr***	1149(88.7)	423(36.8)	0.057	**333(29.0)**	**0.009**	258(22.5)	0.460
**Hepatitis A**	224(17.3)	80(37.7)	0.493	65(29.0)	0.680	50(22.3)	0.863

*Numbers shown in bold are statistically significant (Chi^2 ^test; p < 0.05).

All studied viral infections were associated with each other (data not shown); however, comparing the logistic models using all infections in the same model or with each infection in separate models, there was no significant change in the ORs for the 3 infection-outcome associations, so we included all infections in the same model. There was not important heterogeneity of effect for any of the variables studied using multiplicative interaction terms between each infection and gender in relation to the outcomes (Data not shown).

### Associations of viral infections with anti-IgE antibodies to environmental allergens

Among potential risk factors, only gender was significantly associated with specific IgE using a cut-off of IgE ≥ 0.70 (boys > girls, Table 1). No association was found between viral infections and the presence of specific IgE to environmental allergens using either cut-off (≥0.35 and ≥0.70) (Table 1 and 2).

**Table 2:** Logistic regression analyses of association between seropositivity to common viral infections of childhood and the presence of aeroallergen-specific IgEs using cut-offs for specific IgE of ≥0.35 and ≥0.70 kU/L

Infection by N = 1296	*sIgE ≥0.35 (n = 644/49.7%)			sIgE ≥ 0.70 (n = 489/37.7%)		
	
		OR (95% C.I.)			OR (95% C.I.)	
	n (%)/N	Crude	Adjusted**	n (%)/N	Crude	Adjusted**
**HSV**						
Negative	298(51.0)/584	1	1	235(40.2)/584	1	1
Positive	346(48.6)/712	0.91 (0.73; 1.13)	0.94 (0.75; 1.18)	254(35.7)/712	0.82 (0.66; 1.03)	0.88 (0.69; 1.11)
**VZV**						
Negative	357(50.4)/708	1	1	273(38.6)/708	1	1
Positive	287(48.8)/588	0.94 (0.75; 1.17)	0.93 (0.74; 1.17)	216(36.7)/588	0.93 (0.74; 1.16)	0.93 (0.74; 1.17)
**EBV**						
Negative	80(54.4)/147	1	1	66(44.9)/147	1	1
Positive	564(49.1)/1149	0.81 (0.57; 1.14)	0.82 (0.58; 1.17)	423(36.8)/1149	0.71 (0.51; 1.01)	0.75 (0.53; 1.07)
**HAV**						
Negative	531(49.5)/1072	1	1	409(38.2)/1072	1	1
Positive	113(50.4)/224	1.04 (0.78; 1.38)	1.07 (0.80; 1.44)	80(35.7)/224	0.90 (0.67; 1.22)	0.94 (0.69; 1.28)

*Aeroallergen-specific IgE; **Adjusted for gender and age.

### Associations of viral infections with skin prick test (SPT) for environmental allergens

Only gender was significantly associated with SPT, being more frequent in boys. A negative association of SPT with both HSV and EBV infections were observed (unadjusted, OR = 0.64; CI = 0.51-0.82 and OR = 0.63; CI = 0.44-0.89, respectively; adjusted for age and sex, OR = 0.66; CI = 0.51-0.84 and adjusted OR = 0.69; CI = 0.48-0.99, respectively). No associations were found between SPT and HAV and VZV infections (Table 1 and 3).

**Table 3:** Logistic regression analyses of associations between viral infections and skin prick test reactivity (SPT) for at least one aeroallergen

Infection by N = 1296	SPT ≥0.3 mm		
	n = 391 (30.2%)		
	
	n (%)/N	OR (95% C.I.)	
		Crude	Adjusted*
**HSV**			
Negative	206 (35.3)/584	**1	**1**
Positive	185 (26.0)/712	**0.64 (0.51; 0.82)**	**0.66 (0.51; 0.84)**
**VZV**			
Negative	220 (31.1)/708	1	1
Positive	171 (29.1)/588	0.91 (0.72; 1.15)	0.89 (0.70; 1.14)
**EBV**			
Negative	58 (39.5)/147	**1**	**1**
Positive	333 (29.0)/1149	**0.63 (0.44; 0.89)**	**0.69 (0.48; 0.99)**
**HAV**			
Negative	326 (30.4)/1072	1	1
Positive	65 (29.0)/224	0.94 (0.68; 1.28)	1.01 (0.73; 1.40)

*Adjusted for gender and age. **Bold values are statistically significant (p < 0.05).

### Association of viral infections with asthma

No infection was significantly associated with asthma in either univariate or multivariate analyses. Moisture and mold on the walls and daycare attendance were significantly associated with asthma in univariate but not in multivariate analysis (Tables 1 and 4). No association was found between the viral infections and either atopic or non-atopic asthma using atopic non-asthmatic or non-atopic non-asthmatic children as comparison groups, respectively (Table 5).

**Table 4:** Logistic regression analyses of associations between viral infections and asthma

Infection by	Asthma		
	n = 295 (22.8%)		
	
N = 1272	n (%)/N	OR (95% C.I.)	
		Crude	Adjusted*
**HSV**			
Negative	137 (23.5)/584	1	1
Positive	158 (22.2)/712	0.93 (0.72; 1.21)	1.02 (0.77; 1.35)
**VZV**			
Negative	163 (23.0)/708	1	1
Positive	132 (22.4)/588	0.97 (0.75; 1.26)	1.09 (0.82; 1.43)
**EBV**			
Negative	37 (25.2)/147	1	1
Positive	258 (22.5)/1149	0.86 (0.58; 1.28)	0.81 (0.53; 1.23)
**HAV**			
Negative	245 (22.9)/1072	1	1
Positive	50 (22.3)/224	0.97 (0.69; 1.37)	0.99 (0.69; 1.42)

*Asthma defined as at least 1 case of 4 or more episodes of wheeze within the last 12 months, waking up in the night with shortness of breath, wheeze post-exercise or asthma diagnosed by doctor **Adjusted for gender, age, parental asthma, mother's education and cat at home.

**Table 5:** Polytomous logistic regression analyses of associations between viral infections and asthma

N = 1296	Non-atopic asthmatic N = 154		Atopic asthmatic N = 141		
	
	n (%)	OR (C.I)*	n (%)	OR (C.I)*	OR (C.I)**
**HSV**					
No (n = 584)	70(20.1)	1	67(19.4)	1	1
Yes (n = 712)	84(18.3)	0.96(0.66; 1.40)	74(16.5)	0.94(0.64; 1.38)	1,15(0,76; 1,73)
**VZV**					
No (n = 708)	86(19.8)	1	77(18.1)	1	1
Yes (n = 588)	68(18.3)	1.06(0.74; 1.54)	64(17.4)	1.04(0.71; 1.52)	1,15(0,77; 1,74)
**EBV**					
No (n = 147)	17(21.0)	1	20(23.8)	1	1
Yes (n = 1149)	137(18.9)	0.82(0.46; 1.48)	121(17.0)	0.64(0.37; 1.13)	0,87(0,48; 1,57)
**HAV**					
No (n = 1072)	124(18.7)	1	121(18.3)	1	1
Yes (n = 224)	30(20.8)	1.20(0.75; 1.92)	20(14.9)	0.83(0.49; 1.41)	0,79(0,45; 1,39)

*Odds ratio for comparison with non-atopic non-asthmatics. Adjusted for gender, age, parental asthma, mother's education and cat at home.

** Odds ratio for comparison with atopic non-asthmatics. Adjusted for gender, age, parental asthma, mother's education and cat at home.

## Discussion

In our study, we found no association between the HSV infection and the presence of aerollaergen-specific IgE or asthma; however, a significant negative association was observed with SPT in multivariate analyses, indicating that exposures to this infection may be associated with suppression of immediate hypersensitivity responses. The immune response of allergic diseases is typically Th2; thus, it is possible that pathogenic or non-pathogenic organisms (or their molecules) that suppress this response can induce protection from allergies. Among the known factors that can suppress a Th2 immune response, one can highlight Th1 and T-regulatory (T-reg) cells stimulators. *In vitro *studies have shown that HSV DNA can induce tumor necrosis factor (TNFα) and interleukin (IL)-6, a Th1 immune response, and release interferon (IFN)-γ [19]. Matricardi and collaborators [20], studying a North American population, showed that HSV protected against asthma and hay fever. Janson and colleagues [12], studying young adults in Iceland, Sweden and Estonia, showed that HSV was negatively associated with atopy.

Our data showed that EBV was negatively associated with skin reactivity. EBV has a different mechanism of protection of allergic diseases. It induces T-regs cells as an escape mechanism from the immune system, and although this mechanism has not been well elucidated, it seems to involve an elevation of IL-10 and transforming growth factor (TGF)-β. Wingate and collaborators [21] and Marshall and collaborators [22] found in the EBV genome a well-preserved sequence homologous to IL-10, which may be influencing this immunomodulation [23]. Several studies have reported EBV associated with protection of atopy [13,24].

In relation to HAV infection, our data, as well as others in the literature [12,25,26] showed no evidence of its association with the study outcomes. In addition to being an indicator of poor hygiene conditions, the HAV can also have an immunoregulatory role in reducing asthma in its hosts. Polymorphism of a receptor (TIM-1) used by the hepatitis A virus to invade cells may be related to allergy [27,28]. Some studies have shown that HAV infection is negatively associated with allergic diseases [11,20,29,30]. This difference in findings in different geographic regions indicates that environmental and genetic factors may be influencing the ability of this virus to immunomodulate atopy and allergy or not.

In our study, there was no evidence that VZV infection was associated with the modulation of allergic responses or disease. However, Zhang and colleagues [31] and Janson and colleagues [12] showed that lymphocyte cultures stimulated with this virus could enhance the production of Th1 cytokines. Nilsson and colleagues [13] have also not find any association of this infection with atopy and allergic symptoms in children.

Although in this study EBV and HSV were inversely associated with SPTs, there was no association of these viral infections with specific IgEs against common allergens either using 0.35 or 0.70 kU/L as cut-offs. One possible explanation for these findings is that the presence of EBV, which had high prevalence (88.9%) in our population, stimulated the production of IL-10, thereby preventing mast cell degranulation, even in the presence of IgE and thus decreasing SPT reactivity in children with elevated sIgEs. Another possible explanation is that our population, in contrast to affluent countries, is constantly infected and re-infected by helminths, which have been associated with elevated levels of IgE levels to aeroallergens due to possible cross-reactivity between parasite antigens and aeroallergens [18]. Possibly, as significant component of the specific IgE detected by Immunocap assays are in fact cross-reactive antibodies, which may not have enough affinity for the aeroallergen molecules and therefore do not crosslink in the FCεRII, a necessary step for the occurrence of mast cell degranulation.

The clinical diagnosis of asthma is difficult in pre-school children. Most of the epidemiological studies use symptoms of asthma (wheezing in the last 12 months) or doctor's diagnosis of asthma as the indicators. In the current study we screened all children with a history of wheezing in the last 12 months with a few other questions aiming to increase the specificity of our criteria for asthma: diagnosis of asthma ever, wheezing with exercise; ≥ 4 episodes wheezing, waking up night because of wheezing. They had to have at least one extra positive answer to the queries. Although we suppose our criteria are more accurate than the usual epidemiological definitions, we acknowledge the possibility some of the children we classified as having asthma may have transient wheezing. For that reason we made an analysis excluding children younger than 6 years, the only finding that differ from the previous analysis was the association of EBV infection with specifc IgE which was borderline in the whole children population analysis and became statistically significant but without differ the odds ratio in the analysis with children above 6 year old.

Many studies in the literature have shown negative associations between viral infections and asthma [12-14]; however, there was no strong evidence that the viral infections studied in our population mediate the development of asthma. Pearce and collaborators [32] showed that the positive association between SPTs and asthma is lower in developing countries than in developed countries. Given the large amount of non-atopic asthma found in the children studied herein [33], this may be a possible explanation for the absence an association between asthma and any of the studied viral infections. Furthermore, our data indicate the absence of association of the viral infection with asthma is not restricted to non-atopic asthma, but also to atopic asthma.

## Conclusion

Our data provide further evidence that common viral infections of childhood are associated with an attenuation of immediate hypersensitivity responses in children but not clinical disease. Given that most asthma is not associated with atopy in our study population, the data indicate that factors other than infection should be investigated as causing asthma in populations living in non-affluent countries.

## Study Limitation

The current work is based solely on serology and does not take into account the age of infection or chronicity of infection. Another limitation is its inability to establish causality, since it is a cross-sectional analysis in which there is no information on the temporal sequence between exposure and outcome.

## Competing interests

The authors declare that they have no competing interests.

## Authors' contributions

RVV- carried out the laboratory assays and the statistical analysis and drafted the text. SC- Supervised the field work and the statistical analysis; VCCD - carried out the laboratory work; AC, PJC and LCR - helped in the work design and reviewed the manuscript; MLB - coordinated the SCAALA project, helped in the work design. NMAN - designed the work, coordinated the laboratory work and reviewed the text. All authors have read and approved the final manuscript.

## Pre-publication history

The pre-publication history for this paper can be accessed here:

http://www.biomedcentral.com/1471-2466/11/24/prepub
